# Pristine Inner Experience and Descriptive Experience Sampling: Implications for Psychology

**DOI:** 10.3389/fpsyg.2017.02170

**Published:** 2017-12-12

**Authors:** Leiszle R. Lapping-Carr, Christopher L. Heavey

**Affiliations:** Department of Psychology, University of Nevada, Las Vegas, Las Vegas, NV, United States

**Keywords:** pristine inner experience, inner experience, introspection, descriptive experience sampling, methodology

## Abstract

Pristine inner experience is that which is directly present in awareness before it is distorted by attempts at observation or interpretation. Many psychological methods, including most introspective methods, attempt to measure some aspect of pristine inner experience (thoughts, feelings, mental imagery, sensations, etc.). We believe, however, that these methods produce unspecifiable combinations of pristine inner experience, beliefs about the self, beliefs about what inner experience should be like, inaccurate recollections, miscommunications, and other confounding influences. We argue that descriptive experience sampling (DES) can produce high fidelity descriptions of pristine inner experience. These descriptions are used to create idiographic profiles, carefully crafted, in-depth characterizations of the pristine inner experience of individuals. We believe these profiles, because they are built from moments apprehended via a method that confronts the challenges inherent in examining inner experience, are uniquely valuable in advancing the science of inner experience and psychology broadly. For example, DES observations raise important questions about the veracity of results gathered via questionnaires and other introspective methods, like casual introspection. DES findings also provide high fidelity phenomenological data that can be useful for those developing psychological theories, such as theories of emotional processing. Additionally, DES procedures may allow clinicians and clients to practice valuable skills, like bracketing presuppositions and attending to internal experiences. This paper will describe difficulties inherent in the study of pristine inner experience and discuss implications of high fidelity descriptions of pristine inner experience for psychological research, theory development, and clinical practice.

## Introduction

Psychology, as a science and a profession, relies heavily on introspection. Introspection can be defined as “looking into our own minds and reporting what we discover” (Boring, 1953). Using this definition, introspection includes any effort made by research participants or psychotherapy clients to reflect on their personal, inner experiences and describe that experience, whether through dialog, written summary, or rating scale (Boring, 1953; Clegg, 2012). Questionnaires that ask participants to report on thoughts or feelings require introspection. Sensation research, such as threshold discrimination tasks, require introspection. Cognitive-behavioral treatment often requires introspection (e.g., reporting moods and thoughts, identifying and challenging cognitive distortions, etc.). Almost all forms of therapy employ some form of introspection (e.g., Tell me how you feel about that, etc.). Although behavioral research and interventions are integral to psychology, a science without the mind, and thus a science without introspection, would be incomplete.

## Pristine Inner Experience

Introspection is aimed at understanding first-person, private phenomena, what we call pristine inner experience. Pristine inner experience is that which is directly present in awareness before it is distorted by observation or interpretation (Hurlburt, 2011). It is “pristine in the same sense as we would say a forest is pristine…. Pristine does not necessarily mean ‘clean’ or ‘tranquil’; much of a pristine forest is mucky, bloody, brutal and so on” (Hurlburt, 2011, p. 2). It is not pure, but rather is untouched. Pristine inner experience is often conflated with other things, like beliefs about the self or expectations of what experience should be. These may be valuable pieces of information, but they are not pristine inner experience. If our goal in using introspection is to gain high fidelity descriptions of pristine inner experience, then we must carefully distinguish pristine inner experience from all else.

Gathering high fidelity descriptions of pristine inner experience is difficult (Hurlburt, 2011). People, in general, do not have practice apprehending or describing inner experience. Unlike language for external events, people do not have opportunities to develop and shape their language for inner experience (Skinner, 1974; Hurlburt and Heavey, 2001). To communicate carefully about inner experience, participants and researchers must have an opportunity to refine and clarify their language with each other. Both must collaboratively work toward a mutual understanding (Grice, 1975). For example, when an individual says they feel sad, they may be referring to feeling an empty pit in their stomach, or a heaviness in their arms and legs, or a mental feeling that does not exist in their body, or they may be thinking about a sad event, but not have a direct experience of a feeling at the moment in question. Without a careful conversation, it would be impossible to distinguish these experiences.

Another difficulty of studying pristine inner experience are the presuppositions, or assumptions, that both researchers and participants have about what pristine inner experience is in general or what it should be in particular situations/for particular people (Hurlburt and Heavey, 2006; Hurlburt, 2011). For example, societal expectations that men have less frequent and weaker experiences of feelings than women seem to result in participants reporting consistent with this belief when asked to reflect on their experience over an extended period of time. But men and women do not report significantly different frequencies of feelings when they are asked to report on momentary experiences (e.g., LaFrance and Banaji, 1992). Also, when researchers exclusively ask about a particular experiential phenomenon (e.g., negative feelings), participants are likely to identify negative feelings, leading to an overreporting of negative feelings (Nisbett and Wilson, 1977; Tourangeau, 2000). A research method that is aimed at pristine inner experience should include an explicit denial of any preference for certain experiential phenomena and directions to the participant to try and simply describe what was present in their experience, putting aside (or bracketing^1^) presuppositions (Hurlburt, 2011; Hurlburt and Heavey, 2015). Only by recognizing the importance of bracketing presuppositions are participants and researchers able to work toward obtaining high fidelity descriptions of pristine inner experience.

When participants are asked to reflect and report on their inner experiences, they are often asked to consider a broad time frame. For example, many depression questionnaires use the diagnostically convenient 2-week time frame (e.g., the Patient Health Questionnaire-9; Kroenke et al., 2001). This time frame accesses participant’s beliefs about herself rather than her experience. Studies have shown a significant discrepancy between momentary reports on experience and generalized reports of the same time frame (Robinson and Clore, 2002b). This difference between reports stems from participants drawing on their semantic knowledge, or what they believe to be true about themselves, when they are asked about experience over a broad time frame. When asked about experience at a particular moment, participants will draw on their episodic knowledge, or what they just experienced (Robinson and Clore, 2002a). In order to adhere to pristine inner experience, observations must be tied to specific moments to ensure reliance on episodic, as opposed to semantic, memory. Momentary reporting serves the double purpose of minimizing retrospective demands and reducing the opportunity for presuppositions and beliefs to influence reports.

In summary, three important constraints to studying pristine inner experience are language clarity, bracketing presuppositions, and momentary reporting. Many additional constraints exist (see Hurlburt, 2011). Introspective methods may address some of these concerns, but if we want to gain understanding of pristine inner experience, then our method must work to address all these concerns. Otherwise, the results will be some indefinable mixture of miscommunications, beliefs about inner experience or the self, memory errors, and pristine inner experience (Hurlburt, 2011; Hurlburt and Heavey, 2015). We have found that iterative training is required to address these concerns. Only by meeting repeatedly and discussing the specifics of language, the presence of presuppositions, and just what we mean by “momentary” are participants able to progressively learn to cleave to the moment, apprehend their pristine experience, and describe it in high fidelity (Heavey et al., 2010; Hurlburt, 2011; Heavey, 2012; Hurlburt and Heavey, 2015).

## Descriptive Experience Sampling

Descriptive experience sampling (DES) is an introspective method that was designed to confront these concerns in order to describe pristine inner experience in high fidelity. DES uses a randomized beeper that participants wear in their everyday environment to cue participants to attend to their inner experience at a precise moment. Participants collect about six samples of their inner experience (over approximately 3 h) and take part in an expositional interview within 24 h (the interview is scheduled first, and the participant samples their experience at their convenience). This sampling-then-interview procedure is repeated multiple times, ranging from 4 to 10 days, depending on the study (Hurlburt and Heavey, 2006; Heavey et al., 2010).

During the expositional interview, participants collaboratively work with investigators to clarify everyone’s language and bracket everyone’s presuppositions about the participant’s pristine inner experience. Participants are iteratively trained to apprehend their experience as it was caught in flight by the beep. The investigators always start with the question “What, if anything, was present in your experience at the moment of the beep?” Follow-up questions are all aimed at clarifying the characteristics of the participant’s pristine inner experience. (For a more detailed discussion of the interview, please see Hurlburt and Schwitzgebel, 2007; Hurlburt, 2011.)

After each interview, the investigators write contemporaneous descriptions of each sampled moment. The written descriptions are reviewed by all investigators present at the interview and revised as necessary. The goal is not necessarily consensus, but rather to reflect the pristine inner experience in high fidelity. When pristine inner experience is unclear and messy, the written descriptions will reflect this characteristic. Disagreements between investigators are included in the written descriptions. After the final expositional interview, the investigators meet and discuss the salient characteristics of the participant’s experience that emerged over the sampling days. After the meeting, each investigator independently writes a brief description of the salient characteristics. One investigator then reviews each investigators’ understanding of the salient characteristics and writes an idiographic profile of the participant’s pristine inner experience, including the frequency of different phenomena and the qualitative nature of those phenomena. The idiographic profile is reviewed again and edited by the rest of the investigators. This lengthy process helps the investigators bracket their presuppositions and produce carefully considered, high fidelity descriptions of pristine inner experience. (For a more detailed description of the DES method, please see Hurlburt and Heavey, 2006; Heavey et al., 2010; Hurlburt, 2011; Heavey, 2012.)

Using DES, we have identified five experiential phenomena that occur frequently across participants, termed the “5FP” for “5 frequent phenomena” (Heavey and Hurlburt, 2008; Kühn et al., 2014). We have seen the 5FP in a variety of populations, both psychiatric [different studies of patients diagnosed with depression (Lefforge, 2010), bipolar disorder (Kang, 2015), bulimia nervosa (Hurlburt, 2011), schizophrenia (Hurlburt, 1990), and posttraumatic-stress disorder (Raymond, 2011)] and non-psychiatric undergraduate students (Heavey and Hurlburt, 2008). The 5FP include inner speaking (Hurlburt et al., 2013, 2016), inner seeing (the experience of seeing things mentally), feelings (Heavey et al., 2012, 2017), sensory awareness (Hurlburt, 2009; Hurlburt et al., 2009), and unsymbolized thinking (Hurlburt and Akhter, 2008; Hurlburt, 2009). Across participants, each of the 5FP occur in approximately 25% of sampled moments. However, the frequencies of the 5FP vary drastically by the individual, between 0 and 100% (Heavey and Hurlburt, 2008). Also, many participants experience phenomena that do not fit into the 5FP (e.g., “just doing,” or the experience of being completely absorbed in an activity) and neither are there sharp boundaries that delimit the 5FP. Pristine inner experience is often messy and unable to be neatly categorized. While the 5FP can often be used to succinctly describe some aspects of an individual’s experience, it also very common for aspects of pristine inner experience to be outside these categories, or to include aspects of more than one category. The 5FP are our best attempt at identifying nomothetic themes across participants, but are not always cleanly distinct nor are they fully inclusive of the contents of pristine inner experience (Heavey and Hurlburt, 2008).

## Veracity of Introspective Reports

The idiographic profiles produced by DES provide valuable information about the phenomenology of pristine inner experience that cannot be gleaned through other introspective methods. These results also raise questions about the veracity of the conclusions drawn by other introspective methods. We will use inner speaking as an example, although we could discuss any of the 5FP. Many researchers claim that inner speaking is a predominant part of pristine inner experience (Klinger and Cox, 1987). However, multiple DES studies have consistently found large individual differences in frequency of inner speaking and an across participant average of roughly 25% (Heavey and Hurlburt, 2008; Hurlburt et al., 2013). Further, DES has directly compared questionnaire data on inner speaking to DES results, and found no correlation (Hurlburt and Heavey, 2015; Kelsey, 2016). It is understandable to be suspicious of this finding; however DES identified inner speaking has been validated using fMRI imaging (Kühn et al., 2014; Hurlburt et al., 2016). This suggests that DES is able to identify inner speaking when it is present, and that well-known memory errors and cognitive biases inflate estimates of inner speaking given on self-report questionnaires (Tourangeau, 2000; Robinson and Clore, 2002a,b).

The most common form of introspection is what we can call casual introspection. Casual introspection can be described as engaging in self-initiated, targeted judgments about currently occurring experience (Siewert, 2011). Participants attend to their experience with specific questions in mind (e.g., What are you thinking? How are you thinking it? Why are you thinking it?) and report these experiences, including motivations, causation, analytic thinking, etc. What people observe via casual introspection may well be aspects of human experience, but they are unlikely to be clean or high fidelity observations of pristine inner experience. For example, casual introspection requires analysis, so it’s presumably impossible to employ casual introspection without finding some aspect of analysis. DES occasionally finds pristine inner experiences such as reflecting on or analyzing one’s own thoughts, which we term meta-cognition, but these instances are rare in pristine inner experience (Hurlburt and Heavey, 2006; Heavey and Hurlburt, 2008; Hurlburt, 2011).

Another method of studying experience is the phenomenological interview (Høffding and Martiny, 2016). The goal of the phenomenological interview is (1) to create careful, nuanced descriptions of experience, and (2) to analyze the descriptions using phenomenological theory, yielding insights into the essential structure of experience. There are many similarities between DES and the phenomenological interview (e.g., excluding the causes of phenomena, a collaborative or reciprocal interview, the iterative nature of the analysis). However, the phenomenological interviewer would mostly discard the content of an experience. We believe the content is essential to pristine inner experience, and it is through multiple observations of this content that we can start to build nomothetic themes regarding how experience often presents itself (e.g., the 5FP).

Furthermore, there is experimental support for the notion that engaging in the kind of self-reflection asked for in casual introspection and the phenomenological interview can change the nature of one’s experience. For example, when participants engage in detailed and in-depth introspection about their attitudes, the results are poorer predictors of behavior than simple attitude scales (Wilson et al., 1990; Halberstadt and Wilson, 2008). Similarly, participants asked to track the quality of their learning while engaging in a word-learning task had poorer recall than those participants who did not introspect about their learning (Mitchum et al., 2016). It seems changing the experience is not considered problematic for the phenomenological interviewer (Høffding and Martiny, 2016). However, if the experience is changed, then the results can no longer be considered high fidelity descriptions of pristine inner experience.

## Theoretical Usefulness of Pristine Inner Experience

The idiographic profiles that result from DES provide a high fidelity view of the pristine inner experience of individuals. This view is not available through other methods and can be useful for developing psychological theories. Let’s take emotion development as an example. A commonly accepted theory of emotion development is the Levels of Emotional Awareness model that posits feelings are first recognized in the self before being recognized in others and that certain phenomenological experiences are less advanced than others (e.g., bodily sensations are less sophisticated than blended emotions; Lane and Schwartz, 1987). However, this theory does not consistently correlate with age, as expected, and does not build on other developmental models. A recently proposed perceptual differentiation of feeling model posits that feelings development parallels perceptual development, such that feelings are first recognized in others before the self and that emotional breadth, as opposed to specific phenomena, represents emotional sophistication (Picker, 2017). Besides the support found in correlational data with age, DES results also support this theory. Results from two young adolescents showed that even when there were emotional aspects of their experience (e.g., saying “I am sad” repeatedly), these adolescents rarely, if ever, have a direct experience of a feeling (Hurlburt, 2011). Adults, on the other hand, vary widely in how frequently they experience feelings. Perhaps adults with less emotional breadth have less pristine inner experience that includes feelings (Heavey et al., 2012). Careful observations of pristine inner experience will help develop a more complete understanding of emotional development.

## Clinical Utility of Des Skills

At its essence, DES is an endeavor to understand a single individual’s pristine inner experience as fully as possible. Some characterize psychotherapy similarly: an effort to understand a single individual wholly and completely (McWilliams, 1999). One DES study specifically asked participants about any changes in how they saw themselves and the benefits of participation. Overall, participants reported enjoying the research, learning new things about themselves, and believing it gave them a better understanding of their thoughts and feelings (Turner, 2015).

Cognitive-behavioral therapies often ask participants to attend to and describe their experience at certain times. For example, the classic CBT thought record asks participants to identify a problematic thought, and identify the behaviors and feelings that are associated with it or occurred simultaneously (DeRubeis and Beck, 1988). This requires participants to be able to attend to and identify their thoughts, behaviors, and feelings, which not all clients are able to do. As we’ve explained, DES provides iterative training aimed at improving the skills of participants to apprehend and report on their pristine inner experience in high fidelity; as such it is well-suited to build precisely the observational skills needed for effective CBT.

Descriptive experience sampling also requires both the participant and the investigator to bracket their presuppositions about what inner experience is or should be. DES seeks to observe what is present with no theories about what is good, bad, right, wrong, etc. Clinicians who hear and reflect the experience of the client, without making judgments about what is present, can use this skill to strengthen therapeutic alliance and provide validation. In dialectical behavior therapy and acceptance and commitment therapy, clients are asked to participate in mindfulness exercises to increase their ability to attend to their experience at a particular moment without judgment. The ability to put aside (or bracket) judgment about one’s experience fosters acceptance of what is present and can reduce suffering (Hayes and Smith, 2005; Linehan, 2015).

## Conclusion

Psychology can be understood as the science of the human mind and its functioning. A potentially important aspect of the human mind, and certainly something central to the human experience, is pristine inner experience, that which populates our ongoing awareness. Without carefully designed and conducted introspective research, psychology will lack reliable information about lived, conscious experience. DES aims to gather high fidelity data on pristine inner experience and has shown that it can make important contributions to psychology, including calling into question the veridicality of information about inner experience gathered through questionnaires and other less robust introspective methods (Hurlburt and Heavey, 2015). It has the potential to make many more contributions, such as providing careful observations on which to develop and test theories of emotion development and even potentially increasing the effectiveness of clinical interventions requiring careful introspection or mindful neutrality.

## Author Contributions

LL-C and CH made substantial contributions to the conception of the work, drafting the article and revising it critically for important intellectual content, gave final approval of the version to be published, and agree to be accountable for all aspects of the work in ensuring that questions related to the accuracy or integrity of any part of the work are appropriately investigated and resolved.

## Conflict of Interest Statement

The authors declare that the research was conducted in the absence of any commercial or financial relationships that could be construed as a potential conflict of interest.

## Footnotes

1 “Bracketing” involves attempting to put aside all presuppositions as well as causal discussions and theoretical notions about what is “interesting” or “important” in experience. For more discussion (see Hurlburt and Schwitzgebel, 2011).
